# Non-Epstein-Barr Virus Sinonasal Undifferentiated Carcinoma Presenting as Diplopia and Rhinorrhea

**DOI:** 10.7759/cureus.53185

**Published:** 2024-01-29

**Authors:** Syeda Q Kaifee, Yawar Haq, Birkaran Sadhar

**Affiliations:** 1 Internal Medicine and Ophthalmology, Rochester Regional Hospital, Rochester, USA; 2 Internal Medicine and Ophthalmology, Lake Erie College of Osteopathic Medicine, Erie, USA; 3 Internal Medicine, Semmelweis University, Budapest, HUN; 4 Ophthalmology, Lake Erie College of Osteopathic Medicine, Erie, USA

**Keywords:** orbit malignancy, cerebrospinal fluid rhinorrhea, horizontal diplopia, intracranial mass lesion, snuc, sinonasal undifferentiated carcinoma, nasopharyngeal carcinoma, head and neck cancer, ebv-assocaited carcinoma, ebv

## Abstract

Sinonasal undifferentiated carcinoma (SNUC) is an extremely rare and highly aggressive malignant neoplasm of the nasal cavity and/or paranasal sinuses. SNUC is clinicopathologically distinctive from other tumors but is difficult to study due to its low incidence. There is also very little consensus about the etiology of SNUC, including its association with Epstein-Barr virus (EBV). Treatment modalities include surgery, chemotherapy, and radiation depending on the stage and grading. Herein, we discuss a patient who presented to the emergency department with chronic rhinorrhea and various ophthalmologic symptoms such as flashes, floaters, and diplopia. The patient was later diagnosed with SNUC in the setting of negative serological testing for EBV in addition to his previously concomitant history of bladder cancer. The purpose of this case report is to contribute to the broader literature of SNUC and the specifics surrounding the diagnostic modalities utilized, management, and outcome of non-EBV sinonasal undifferentiated carcinoma in a patient with atypical symptomatology.

## Introduction

First reported in 1986, sinonasal undifferentiated carcinoma (SNUC) is an extremely rare and highly aggressive malignant neoplasm of the nasal cavity and/or paranasal sinuses [1]. It commonly presents as a blockage in the nasal passages, accompanied by headaches and facial discomfort. Additionally, visual issues, protrusion of the eyeball, and deficits in cranial nerves may arise. The symptoms tend to advance rapidly over a period of weeks to months [1,2]. Visual impairment is generally due to compression of the intraorbital optic nerve. It has also been reported that the extension of the tumor through the anterior cranial fossa can cause compression of the optic tracts. Tumor diagnosis via surgical biopsy and tumor extension can be determined via CT or MRI. It is typically diagnosed in later stages due to fairly benign and non-acute presentation [2,3]. Imaging typically shows an advanced lesion within the orbit and expanding to other adjacent structures, and intracranial extension is possible. CT imaging reveals non-calcified masses alongside sinus obstruction, usually with adjacent bony invasion or remodeling. Soft tissue attenuation is seen on non-contrast CT, and contrast imaging displays variable enhancement. However, radiography alone is not enough to differentiate most sinonasal tumors. The pathology report following a surgical biopsy of the tumor offers a more definitive diagnosis. [1]. SNUC typically is a large, fungating tumor with poorly defined margins. Light microscopy shows hypercellular proliferation and various growth patterns with extensive necrosis. Most cells must, by definition, be undifferentiated and pleomorphic [2]. Optimal treatment of SNUC is an area of ongoing interest and has yet to be determined. Different combinations of chemotherapy, adjunctive therapy, and surgical resection have been utilized as there is no universal treatment strategy. Surgical resection should be included when negative margins are deemed feasible [4,5]. While our patient displayed the typical ophthalmologic manifestations such as diplopia and proptosis, he also presented with chronic rhinorrhea later found to be leaking cerebrospinal fluid (CSF). Additionally, our patient had no history of Epstein-Barr virus prior to his cancer diagnosis. Our case offers an atypical presentation, diagnostic indications, and management in a patient found to have SNUC. 

## Case presentation

This patient is a 59-year-old male with a past medical history of hypertension, eosinophilic esophagitis, and malignant neoplasm of the bladder who initially presented with recurrent intermittent vertical diplopia, flashes, and floaters in his left visual field for one month. His diplopia was more significant on awakening and he described mild pain on the outer aspect of the lateral wall of the orbit of the left eye. Evaluation by an ophthalmologist revealed bleeding in the retina and vitreous of his left eye due to age-related changes. The patient was then advised to follow up with his primary care physician. The medication history of the patient was amlodipine 10 mg orally once a day, atenolol 25 mg orally once a day, and omeprazole 20 mg once a day. He has a past surgical history of transurethral resection of the bladder (TURBT). The patient denied associated systemic symptoms including hyperthyroid/Graves-related fatigue, increased respiratory effort, struggling to breathe (myasthenic symptoms), and motor weakness (stroke). A review of the systems was negative except for visual disturbance and tremors.

On focused ophthalmic exam, intraocular pressures were 14 OU and visual acuity was 20/30 on the right eye and 20/40 on the left eye. The remainder of the ophthalmic exam showed no abnormalities. On physical exam, the left eye was slightly more protuberant than the right eye. There was significant horizontal diplopia initially in the left visual field and vertical diplopia in the inferior visual field. All blood and chemistry panels including CBC, BMP, and CMP were all within normal limits at this time. An acetylcholine receptor antibody panel was also ordered to rule out myasthenia gravis as it could present as mono-ocular diplopia. Notably, serologies for Epstein-Barr virus were negative. 

Due to mono-ocular involvement, the possibility of systemic diseases that are more acute and emergent in nature such as stroke, posterior cerebral artery aneurysm, Grave’s disease, central demyelinating disease, and cavernous sinus thrombosis were determined to be less likely. Suspicion for orbital cellulitis was low as the patient denied any pain with extraocular muscle movement and experienced no photophobia. Additionally, retinal and vitreous detachments were deemed unlikely etiologies as their presentation is usually acute in nature, and our patient had been experiencing diplopia for one month.

**Figure 1:**
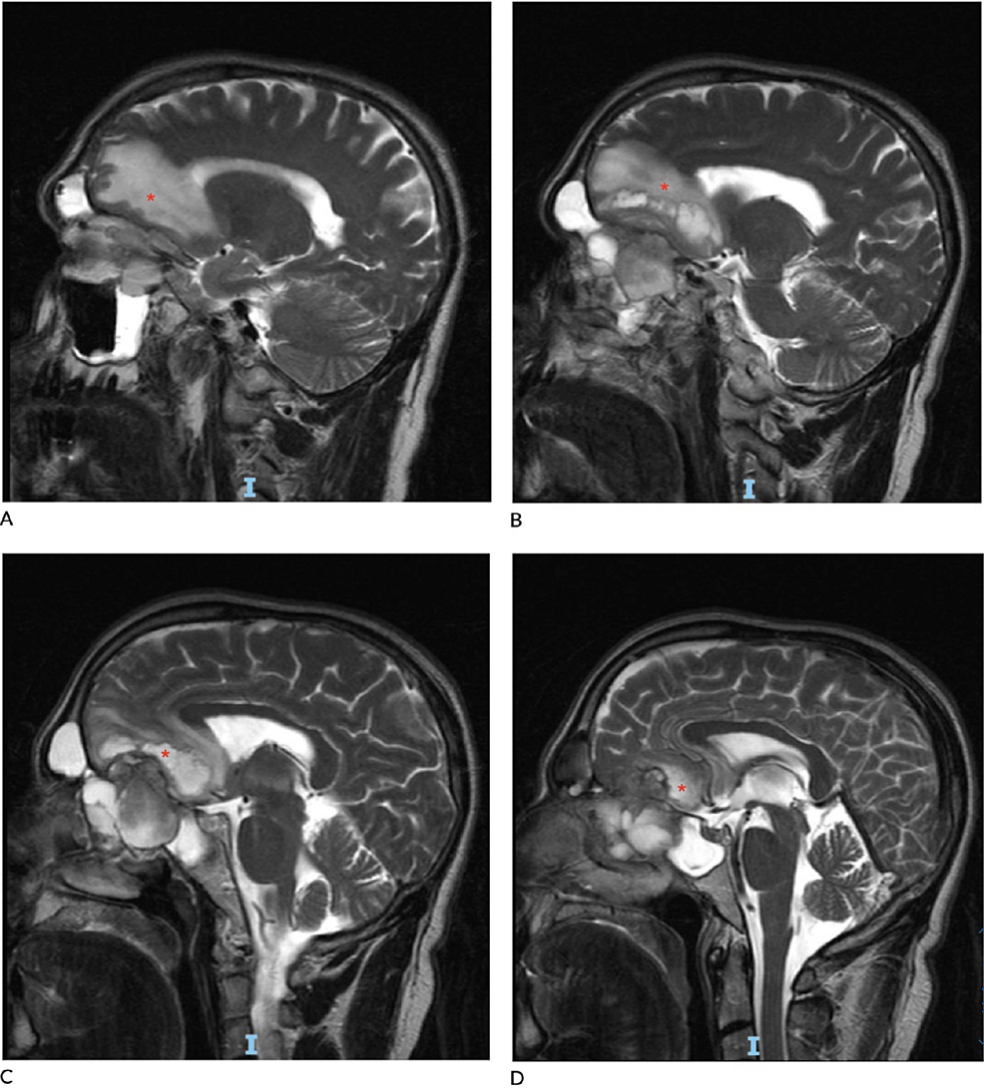
Sagittal MRI of sinonasal mass in ethmoids and extending intracranially, indicated with red asterisk (A-D).

In order to rule out any intracranial and intraorbital pathology, an MRI was determined to be the next step in the evaluation of the orbit and soft tissue structures around the left eye. An outpatient MRI brain showed a nasal mass within the ethmoids with intracranial extension (Figures 1A-1D). The recurrent nature of the diplopia and MRI finding of a nasal mass indicated the need for flexible fiberoptic nasal endoscopy. Findings included no masses, lesions, polyps, or pus on the right side. The left side presented with a fleshy mass filling the middle nasal meatus on the medial-temporal side, but not obstructing the nasal airway. No overlying mucosal ulceration or areas of bleeding. Clear white drainage was present throughout. There was no appreciable change after employing the Valsalva maneuver, including no noticeable pulsations or expression of clear fluid. The mass appeared to extend posteriorly within the ethmoid cavity, leaving the inferior turbinate intact. The nasopharynx was clear and the sphenoethmoid recess contained some edema.

**Figure 2:**
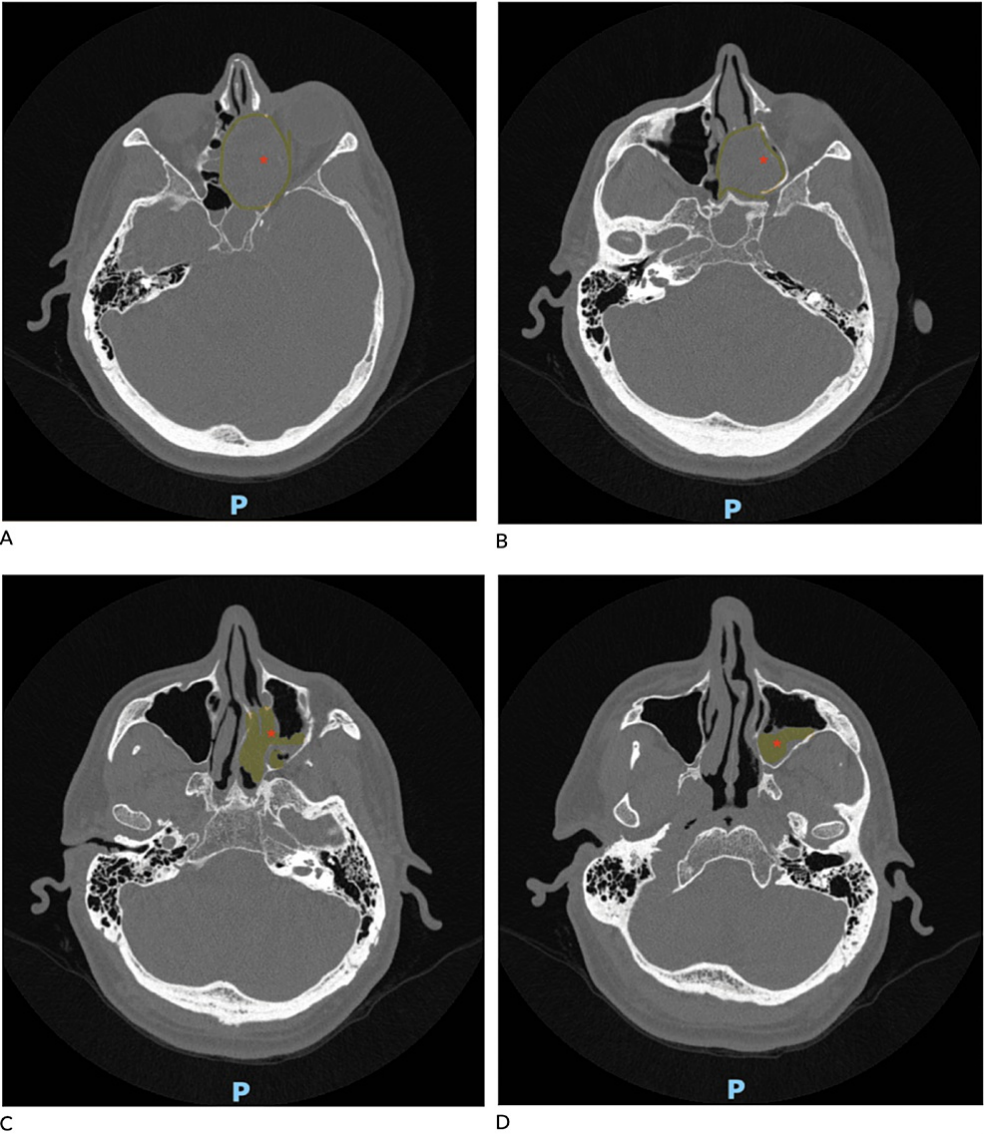
Axial CT sinus landmark without IV contrast from superior to inferior view (A-D). The sinonasal mass is marked with a red asterisk. Yellow shading and outlines indicate orbital and ethmoid sinus invasion of the tumor.

A CT sinus landmark without IV contrast was obtained to properly evaluate the bony anatomy surrounding the nasal mass and determine the course of surgical management (Figures 2A-2D). A complex mass centered in the region of the left ethmoid air cells measuring 4.7 cm in diameter was incompletely evaluated due to the lack of IV contrast and better evaluated from the prior MRI. The mass extended and destroyed the medial left orbital wall, impinging on the orbit as well as the inferior left frontal lobe. The differential diagnosis included squamous cell carcinoma, esthesioneuroblastoma, and atypical infection of fungal etiology. Obliteration of the left ethmoid air cells by the mass resulted in occlusion of the left ostiomeatal unit as well as the frontoethmoidal and sphenoethmoidal recesses, causing opacification of the frontal, sphenoid, and maxillary sinuses. The patient’s rhinorrhea was determined to be cerebrospinal fluid leakage as the nasal mass appeared to be a peripheral enhancing, mostly cystic lesion seemingly originating from the ethmoid sinus and cribriform plate with intracranial extension into the left frontal lobe with some associated edema. 

**Figure 3:**
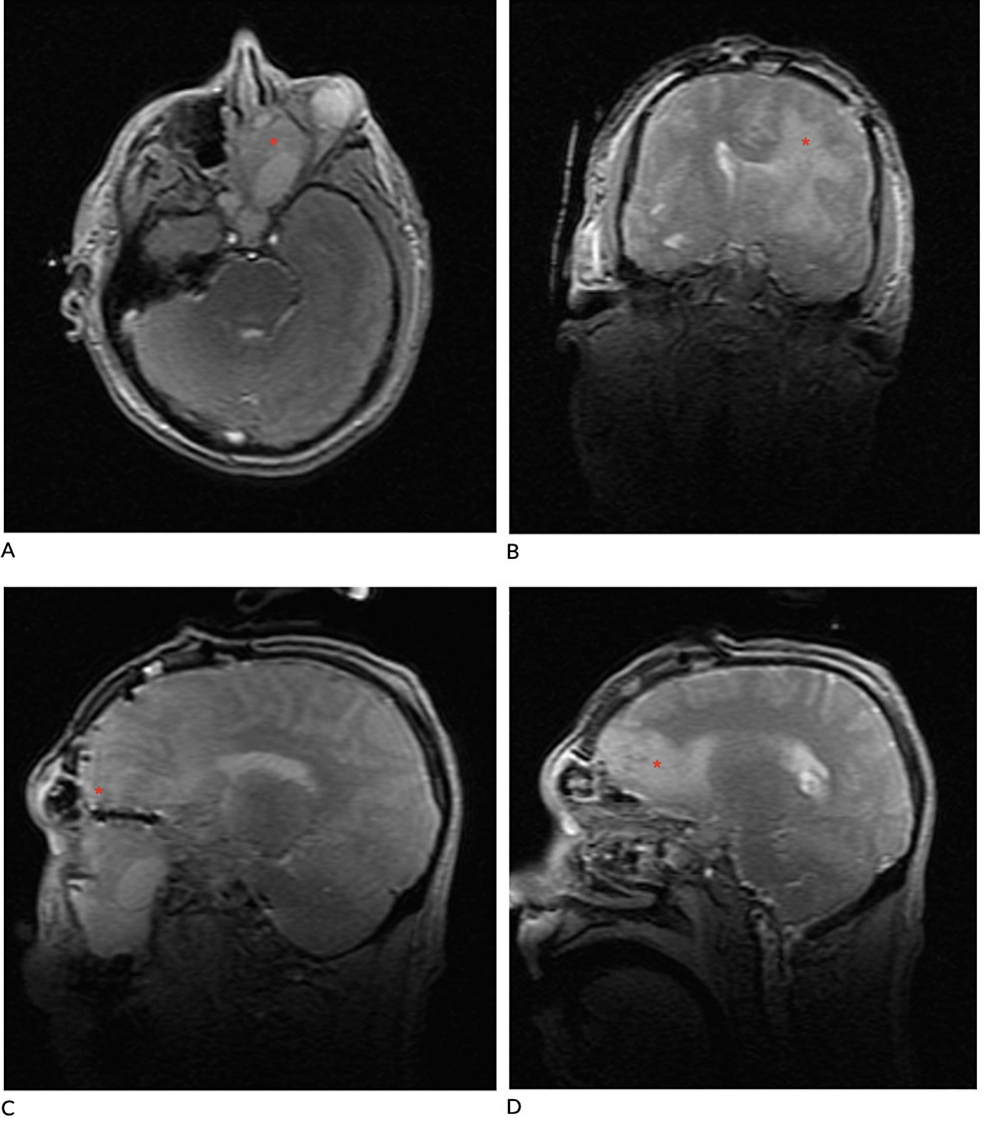
Repeat MRI status post-craniotomy resection of mass. A: axial MRI showing the extra-orbital area of resection of sinonasal mass, indicated by the red asterisk; B: axial MRI showing varying areas of vasogenic edema status post craniotomy, indicated by the red asterisk; C-D: sagittal MRI showing hyperattenuating areas of vasogenic edema consistent with normal changes after complete tumor resection, indicated by the red asterisk.

Due to intracranial involvement, neurosurgery was consulted and an endonasal biopsy was done to identify the mass as either an encephalocele or mucocele versus a cystic tumor. The biopsy revealed a high-grade malignant neoplasm, a favorably sinonasal undifferentiated carcinoma. He underwent bifrontal craniotomy for tumor excision and had a near-total resection of the intracranial portion and repair of the cribriform plate. Repeat MRI revealed complete resection of intracranial extension and likely acute/subacute infarct in the medial left occipital lobe (Figures 3A-3D). He was extubated on postoperative Day 9. The hospital course was complicated due to right upper extremity deep vein thrombosis for which he was given enoxaparin and switched to apixaban prior to discharge. Final management consisted of the radiation oncology recommendation that the patient follow up in three to four weeks. Our patient remained asymptomatic for seven months until he returned with diplopia and obtained an MRI, revealing tumor recurrence. 

## Discussion

SNUC is a rare aggressive neoplasm arising in the nasal cavity and paranasal sinuses and has been reported to have an association with EBV. SNUC typically presents initially with symptoms such as difficulty breathing through the nose and sinonasal pain. With progression, other common symptoms include facial pain, epistaxis, proptosis, and periorbital swelling [1]. Our patient presented with intermittent vertical diplopia and was later found to have bleeding in his left eye. He had slight protuberance of his left eye on later examinations. While earlier literature described the role of EBV in the pathogenesis of SNUC, our patient had negative EBV serologies, supporting the current reports which show that morphologically well-defined tumors have not shown the EBV genome [6]. Cases have been reported to cause diplopia in other patients through mechanisms such as orbital invasion and sixth cranial nerve palsy. In addition, patients who do not have diplopia present mostly with excessive rhinorrhea [6]. Our patient had not received any previous chemotherapy regimens, so rhinorrhea cannot be attributed purely to oncologic therapy in the setting of his previous transitional cell carcinoma. His diplopia presented alongside rhinorrhea, which is also atypical. 

Currently, there is very little known about the etiology of SNUC and the role of Epstein-Barr virus (EBV) in the development of SNUC remains under investigation [7]. A previous study found that EBV had a greater association with SNUC in Asian populations compared to Western populations [8]. However, a more recent study reported no association between EBV and SNUC in 36 Asian patients [7]. Moreover, Cerilli et al. found that the EBV genome was not present in 25 patients with strictly histologically defined SNUC [9]. This discrepancy can possibly be attributed to the fact that the concurrent EBV and SNUC cases were not “true” SNUC as they may have had lymphoepithelial-like features as sinonasal lymphoepithelial carcinoma is associated with EBV [9]. Therefore, our case is most likely a true histologic presentation of SNUC and further supports the literature that there may not be an association between SNUC and EBV.

Additionally, our patient also has a previous history of bladder cancer, however, the incidence of having primary bladder cancer with primary sinonasal cancer is also uncommon, although there are a few reported cases of metastasis between the bladder and sinuses [10,11]. Sinonasal cancer is usually found alongside paranasal cancers, lung cancer, and bone cancer, whereas bladder cancer is found alongside lung, bone, prostate, and liver cancer [12,13]. However, it should be noted that there is currently limited literature to confirm that a history of bladder cancer may be associated with an increased risk of developing sinonasal cancer. One of the important risk factors for both bladder and sinonasal cancer includes occupational exposure to materials such as sheet metal and clothing fibers, both of which were not associated with our patient [14]. Based on a review of previous literature, we believe there is evidence to support that this is a rare presentation of sinonasal undifferentiated carcinoma which did not occur simultaneously with ongoing EBV infection, or with a history of occupational exposure, or previous EBV infection. 

SNUC commonly leads to a poor prognosis as it is frequently detected in advanced stages. A poorer prognosis tends to be associated with neck and distant metastasis and positive resection margins [15]. Chambers et al. established a five-year survival rate of 34.9%, noting a mortality rate 2.5 times higher among individuals aged 70 or above [16]. Khan et al. found the overall five-year survival rate to be 42.2% [4]. The study also highlighted a lower mortality rate among individuals who underwent both surgical resection and chemoradiotherapy compared to those who solely underwent chemoradiotherapy [4]. 

## Conclusions

SNUC, an aggressive tumor of unknown origin, is linked to occupational exposure and was previously thought to involve Epstein-Barr virus infection in disease progression. However, our patient's lack of these associations makes this presentation notably intriguing. Patients typically display symptoms such as diplopia, proptosis, and headache, but can also have an atypical presentation which can include rhinorrhea. The prognosis for SNUC worsens over time if untreated, especially in the presence of metastasis. Hence, it remains crucial for physicians to consider SNUC in patients exhibiting either typical or atypical symptoms, irrespective of their lack of concerning past infection or social history. 
